# Acknowledgment to Reviewers of *International Journal of Neonatal Screening* in 2020

**DOI:** 10.3390/ijns7010004

**Published:** 2021-01-18

**Authors:** International Journal of Neonatal Screening Editorial Office

**Affiliations:** MDPI AG, St. Alban-Anlage 66, 4052 Basel, Switzerland

Peer review is the driving force of journal development, and reviewers are gatekeepers who ensure that *International Journal of Neonatal Screening* maintains its standards for the high quality of its published papers. Thanks to the cooperation of our reviewers, in 2020, the median time to first decision was 16 days and the median time to publication was 39 days. The editors would like to express their sincere gratitude to the following reviewers for their precious time and dedication, regardless of whether the papers were finally published:
Aldirbashi, OsamaLoeber, J. GerardAllewelt, HeatherLorey, FredAlsharhan, HindLund, Allan M.Alves, SandraMandel, HannaBakkeheim, EgilMashima, RyuichiBali, DeekshaMcGill, James J.Barreto, CelesteMei, JoanneBasit, SulmanMellander, MatsBeblo, SkadiMercer, KristinaBessey, AliceMerritt II, J. LawrenceBhattacharjee, ArindamMillington, DavidBonham, JimMoser, Ann B.Botkin, JeffreyMusumeci, OlimpiaBouva, Marelle J.Nährlich, LutzBrockow, InkenNewton, ValerieBurton, BarbaraNiu, Dau-MingBush, Lynn WeinOrsini, JoeCaggana, MichelePadilla, CarmencitaCastilla-Rodríguez, IvanPaton, RobinChakraborty, PraneshPeck, Dawn S.Chen, Pin-WenPena, LorenCicalini, IlariaPetritis, KonstantinosClaahsen-Van-der Grinten, Hedi L.Pitt, JamesClarke, Lorne A.Png, May EeCocho, José A.Ranieri, EnzoCohen, Arieh SierraRaraigh, Karen S.Cordovado, Suzanne KehoeRegelmann, Molly O.Coutinho, Maria FranciscaReuser, Arnold J. J.Currier, RobertRiede, Frank-ThomasDankert-Roelse, Jeannette E.Rinaldo, PieroDavies, GwynethRizzo, WilliamDe Hora, Mark R.Rocha, HugoDe Winter-de Groot, Karin M.Rossi, ClaudiaDel Rosal, TeresaSanchez Russo, RossanaDhiman, VineetSandlers, YanaDluholucký, SvetozárScharfe, CurtElMallah, Mai K.Scheuerle, AngelaEwer, AndrewSchielen, PeterFarrell, Philip M.Sciortino, StanleyFeucthbaum, LisaSeeherunvong, TossapornFichna, MartaServais, LaurentFicicioglu, CanShigematsu, YousukeFingerhut, RalphShintaku, HaruoFukuda, SeijiShone, ScottGasperini, SerenaSingh, RajendraGaviglio, AmySinlcair, GrahamGeppert, JuliaSkov, MarianneGibson, James B.Sola, AugustoGiugliani, RobertoSommerburg, OlafGrange, Dorothy KatherineSontag, MarciGrosse, Scott D.Speiser, Phyllis W.Hagar, ArthurStepien, Karolina M.Hall, KateSuñer, Damian HeineHall, Patricia L.Tajima, GoHamazaki, TakashiTang, HaoHammermann, JuttaTavakoli, Norma P.Heather, NatashaTavartkiladze, GeorgeHeeg, MaximilianTerlizzi, VitoHeld, Patrice K.Therrell, BradfordHowell, RodneyTizzano, EduardoHsuan-chieh, LiaoTluczek, AudreyHwu, Wuh-LiangTomatsu, ShunjiJansen, MarleenTortorelli, SilviaKarlsson, LeifTravert, GeorgesKay, Denise M.Trebušak-Podkrajšek, KatarinaKemp, StephanUrv, Tiina K.Kemper, Alex R.Vergano, Samantha A.Keutzer, JoanVockley, JerryKharrazi, MartinVon Döbeln, UlrikaKim, Mimi S.Walsh, William FrancisKobayashi, HiroshiWanders, RonaldKubaski, FrancyneWebster, DianneKumar, Arun BabuWiesinger, ThomasKuntz, Nancy L.Wiley, VeronicaLaney, Dawn A.Williams, MoniqueLangfelder-Schwind, ElinorWood, TimLatalski, MichałYahyaoui, RaquelLevy, HarveyYamada, KenjiLiao, Hsuan-ChiehYamaguchi, SeijiLin, Hsiang-YuZetterström, Rolf H.Lin, Shuan-Pei


